# Who Are the True Fans? Evidence from an Event-Related Potential Study

**DOI:** 10.1371/journal.pone.0129624

**Published:** 2015-06-09

**Authors:** Qingguo Ma, Jia Jin, Ruixian Yuan, Wuke Zhang

**Affiliations:** 1 School of Management, Zhejiang University, Hangzhou, China; 2 Business School, Ningbo University, Ningbo, China; University of Akron, UNITED STATES

## Abstract

Fans of celebrities commonly exist in modern society. Researchers from social science have been concerned with this problem for years. Furthermore, such researchers have attempted to measure people’s involvement with celebrities in various ways. However, no study measured the degree of addiction to a specific celebrity at the neurological level. Therefore, the current study employed visually evoked event related potentials (ERPs) to examine people’s attitude toward celebrities by comparing different brain activities of fans and non-fans when they were shown a set of photos. These photos include a specific celebrity, a familiar person, a stranger and a butterfly. Furthermore, to examine the validity of the detected neural index, we also investigated the correlation between brain activity and the score of the Celebrity Attitude Scale (CAS), which was a questionnaire used to explore people’s attitude toward celebrities at behavioral level. Two groups of subjects were asked to complete an implicit task, i.e., to press a button when a picture of a butterfly appeared. Results revealed that fans showed significant positive N2 and P300 deflection when viewing the photos of their favorite celebrity, whereas in the non-fan group, the subjects only showed larger P300 amplitude as a response to the celebrity’s photos. Furthermore, a positive correlation between P300 amplitude elicited by the stimuli of a celebrity face and CAS scores was also observed. These findings indicated fan attitude to a specific celebrity can also be observed at the neurological level and suggested the potential utility of using ERP component as an index of fandom involvement.

## Introduction

The notion of fandom was used to understand the linkage between celebrities and their followers in various realms of popular culture, such as film, music, theater, and sport [1, 2]. Fandom widely occurs in contemporary society, and may be regarded as a common activity for individuals to enhance prestige, self-esteem, and social capital [3]. Researchers in the various social sciences have discussed fans, and some studies also focused on determining an effective method to measure fans’ attitude toward celebrities. For example, McCutcheon and colleagues (2002) proposed the absorption–addiction model. In this study, degree of celebrity worship was divided into three levels based on degree of addiction, namely, entertainment-social, intense-personal, and borderline-pathological [4]. In a subsequent study [5], they also developed a Celebrity Attitude Scale (CAS) to define the different levels of worship. However, no study has attempted to find a neural index of fandom, and the neural mechanism of involvement to celebrities has not been a focus.

Many researchers have started to employ neuroscience tools to investigate people’s attitude toward different kinds of people. For example, Vico and colleagues (2010) examined the different affective processing for loved faces, famous people, unknown people, babies, and neutral faces using evidence from peripheral and central electrophysiology. They found that the loved faces resulted in higher valence and arousal, especially for the face of romantic partners, which was reflected by faster heart rate, skin conductance, and zygomatic activity, as well as larger amplitudes of the ERP components P300 and LPP [6]. Furthermore, numerous studies have been conducted to investigate the cognitive process of facial perception and recognition [7,8,9], because the face is a key aspect of emotion communication and personal identity.

Therefore, we hypothesize that people’s attitude toward a specific person may be reflected in their brain responses to his/her photos. In the current study, we considered to evaluate people’s attitude toward a specific celebrity by comparing different groups’ brain activities when viewing the face of a celebrity, a familiar person, and an unknown person.

The current study intended to employ event-related potentials (ERPs) to investigate fans’ and non-fans’ different attitude toward a specific celebrity. The participants were presented with a series of photos, including the face of fans’ favorite celebrity, a stranger, and a learned familiar person, as well as photos of a butterfly. They were asked to complete an implicit task that was unrelated to the faces, i.e., to press the button when the butterfly appeared. After the EEG experiment, the participants were asked to complete the Celebrity Attitude Scale (CAS), which was used to evaluate to people’s attitude to celebrities. We were interested in the different neural processes of fans and non-fans while viewing the photos of the fans’ favorite celebrity, a familiar person, and an unfamiliar person, as well as the correlation between brain activities and CAS scores.

Based on previous studies, we hypothesized that two ERP components may be involved in the current study, namely, N2 and P300. The negative component N2 peaks around 200 ms to 300 ms after the onset of a stimulus, which has been reported to appear in facial processing experiments [8]. Many studies have found that the visual N2 component was related to the deviation from the perception of the target cognitive control and action inhibition processes [10]. For example, Eimer (1993) employed the go/no go paradigm to conduct two experiments, in which the participants were asked to respond to a letter (go stimulus) but not to another (no go stimulus). Results showed that the N2 enhancement elicited by the no go stimulus was larger than that induced by the go stimulus, which reached its maximum in frontal. They suggested that the result was due to the response mismatch and action inhibition [11]. Subsequent studies also explain the larger non-target N2 amplitude as subjects’ inhibition of an anticipated response to the target [12].

Different from the early stage components, the latter positive component P300 was a family of distinct late positive components with divergent distributions over the scalp. This component was sensitive to the different experimental factors, reflecting distinct mental operations [13,14]. The temporal–parietal P300, also known as P3b, has been associated with attention and related to subsequent memory processing [15]. Certain studies reported that P300 amplitude was enhanced while viewing affective pictures, suggesting increased resource allocation to emotionally engaging stimuli [16,17]. Motivationally relevant pictures utilize greater attentional resources than do neutral pictures. Thus, P3 amplitude is interpreted as indexing resource allocation to process emotion stimulus. Additionally, Grasso, D. J and colleagues found that mothers can easily distinguish the faces of their own babies, which elicited larger P300 amplitude than other faces [8]. P300 enhancement was also found in viewing faces of other familiar loved ones (e.g. romantic partner, parents), compared with other’s faces (stranger, learned familiar, even friends) [6,18].

According to the aforementioned findings, we hypothesized that in the current study, fan and non-fan groups may represent different brain activities while viewing the photos of different people, which were manifested in N2 and P300 amplitudes. In the fan group, compared with the picture of the stranger and familiar people, smaller N2 amplitude accompanied by larger P300 amplitude would be elicited when processing the photos of the celebrity. By contrast, in the non-fan group, the photos of the celebrity would not present notable amplitude differences from the other two sets of photos.

## Methods

### Ethics statement

The study was approved by the Internal Review Board of Zhejiang University Neuomanagement Lab. The rights and privacy of the participants was observed. Written informed consent was obtained from all participants prior to the commencement of the experiment. All the participants received monetary compensation for their time.

### Participants

Thirty right-handed healthy graduates and undergraduates between 19 and 25 years old (21.57 ±1.57) were enrolled into the current study. Subjects in the fans group were college students recruited from the fan community of a famous Chinese singer, whereas non-fan group participants were students from Zhejiang University. All subjects were screened using a questionnaire, in which they had to report their liking for and evaluation of the singer. The liking for the celebrity was reported using a seven-point Likert scale. With a score higher than or equal to 4 and a positive evaluation of the celebrity, a subject from the fans community could join the subsequent ERPs experiment as a member of the fan group. Similarly, the non-fan group subjects were those enrolled from the university, and reported a score less than 4 and a neutral evaluation of the celebrity. Each group comprised 3 males and 12 females, because most fans were female. All participants were native Chinese speakers, had normal or corrected-to-normal vision, and did not have any history of neurological disorder or mental disease.

### Stimuli and materials

The experiment was composed of four blocks, with each block including 40 trials, totaling 160 trials. Participants were shown four different kinds of pictures, including photos of a celebrity, a familiar person, an unfamiliar person, and a butterfly. Each category was composed of 40 trials. The experiment used 20 different photos of the selected Chinese famous singer and collected 20 different photos of two female models. Both of the two female models had similar age, hair, and other physical attributes with the celebrity. The perspective, posture, and expression were almost the same among the three different sets of photos. One of the two female models was chosen to be the learned familiar person and counterbalanced between subjects. The photos and basic information (including name and identity) of the model were sent to the participants via e-mail. The participants were instructed to be familiar with the model within the following week. The participants were also asked to complete three tests on the information of the learned familiar person during the week. Before the experiment started, another test was conducted, and only those who successfully selected the model’s photo from 30 different photos can participate in the formal experiment.

The photos, with 300×300 resolution, only show the shoulder and above on a black background. After the experiment, the participants were asked to complete the Celebrity Attitude Scale (CAS). The questionnaire was translated into Chinese by five graduate students and then translated back to English. This procedure was repeated three times to confirm correct translation.

In the current experiment, we compared the ERPs generated by the participants familiar with the famous celebrity in response to familiar and unfamiliar faces to rule out the impact of familiarity of faces, similar to previous studies (e.g.,[8,19]). The reason we chose to control familiarity by including a newly learned face instead of an acquaintance was to keep the same degree of familiarity between subjects. Using pictures of acquaintances would lead to difficulty in controlling the time the subjects had known the acquaintance and their intimacy degree. Thus, we would have little control over the degree of familiarity with the acquaintance.

### Experimental task and procedure

Before the experiment started, subjects were asked to finish a questionnaire about their liking for and evaluation of the selected famous singer to further confirm if they were fans. Each participant was then asked to select the photo of the familiar person from 30 different photos. Only those who picked the right photo could continue to the subsequent EEG experiment.

In the EEG experiment, the participants were comfortably seated in a sound-attenuated room 100 cm away from a computer-controlled monitor. The stimulus was presented sequentially in the center of the CRT computer screen (6.2° × 6.2°). The participants were provided a keypad for responses. Each trial began with a fixation cross on a black background presented for 500 ms. After another 500 ms, a picture appeared for 1 second. The four sets of photos were displayed in random order. Between each trial was a 1-second interval. Stimuli and recording triggers were presented using Stim2 (Neurosoft Labs, Inc. Sterling, USA). Considering that the current study was an implicit design, participants were instructed to press the button as soon as the picture of a butterfly appeared. The experiment started after 10 training trials. After the EEG experiment, the subjects were asked to complete the Chinese version of the CAS.

### Behavioral data analysis

Behavioral data was recorded using Stim2. We compared the actual time of identification of the butterfly and reaction time between groups. Therefore, independent sample analysis was conducted between groups.

### ERP recording and data analyses

Electroencephalogram (EEG) was recorded (band-pass 0.05–70 Hz, sampling rate 500 Hz) with Neuroscan Synamp2 Amplifier (Scan 4.3.1, Neurosoft Labs, Inc. Virginia, USA) using an electrode elastic cap with 64 Ag/AgCl electrodes following the international 10–20 system. A cephalic (forehead) location was used as ground and the left mastoid was used for reference. Data was transferred to the average of the left and right mastoids reference off-line. Electrooculogram (EOG) was recorded from electrodes placed at 10 mm from the lateral canthi of both eyes (horizontal EOG) and above and below the left eye (vertical EOG). EOG artifacts were corrected offline for all subjects. The experiment started only when electrode impedances were maintained below 5 kΩ.

Data were analyzed by using Neuroscan 4.3.1. EOG artifacts were corrected using the method proposed by Semlitsch and colleagues [20]. Trails containing amplifier clipping bursts of electromyography activity or peak-to-peak deflection exceeding ±80 μV were excluded. The recorded EEGs over each recording site for every participant were averaged separately within each of the experimental conditions. The averaged ERPs were then digitally filtered using a low pass filter at 30 Hz (16 dB/octave).

EEG recordings were segmented for the epoch from 200 ms before onset of target appearing on the video monitor to 800 ms after the onset, with the first pre-targets at 200 ms as a baseline. For further analysis, data were composed separately based on different subjects and different stimuli conditions. Each of the two subject groups had three conditions, namely, Unfamiliarity, Familiarity, and Celebrity. Based on visual observation of the grand average waveforms and based on Picton (2000)[21], we chose the time window from 180 to 240 ms after the onset to analyze the mean amplitude of N2 and selected six electrodes (F3, FZ, F4, FC3, FCZ, and FC4) in the frontal-central area. A mixed design ANOVA comprising 2 (groups: fans and nonfans) × 3 (three conditions: Unfamiliarity, Familiarity, and Celebrity) × 6 (six electrodes: F3, FZ, F4, FC3, FCZ, and FC4) was conducted using the subject group as a between-subjects factor.

Similarly, we chose the time window 440 ms to 640 ms after the onset to analyze the mean amplitude of P300, respectively. Six electrodes (CP3, CPZ, CP4, P3, PZ, and P4) in the central-parietal area were selected for statistical analysis. We also conducted a mixed design ANOVA comprising 2 (groups: fans and nonfans) × 3 (three conditions: Unfamiliarity, Familiarity, and Celebrity) × 6 (six electrodes: CP3, CPZ, CP4, P3, PZ, and P4) using the subject group as a between subjects factor.

If a significant interaction effect occurred with faces and groups, a simple effect analysis was separately conducted in each group. Pearson correlation was also conducted between the mean amplitudes of P300 and the CAS scores in the three dimensions separately. The Greenhouse–Geisser [22] correction was applied in all statistical analyses when necessary (uncorrected df are reported with the ε and corrected p-values), and the Bonferroni correction was used for multiple paired comparisons.

## Results

### Behavioral results

The accuracy of the task was 100% for the two groups. The reaction time of the two groups was not significantly different, *T (28) = 0*.*953*, *P > 0*.*05*.

### Event-related potentials results

#### N2 results

A 2×3×6 mixed design ANOVA showed a significant main effect for different observation faces, *F (2*, *56) = 15*.*12*, *p < 0*.*001*, for N2. However, the main effect of subject groups was not observed, *F (1*, *28) = 1*.*56*, *p > 0*.*05*. Additionally, a notable interaction effect with face and subject group was observed, *F (2*, *56) = 3*.*76*, *p < 0*.*05*. Therefore, a simple effect analysis was conducted in each group separately.

**Fig 1:**
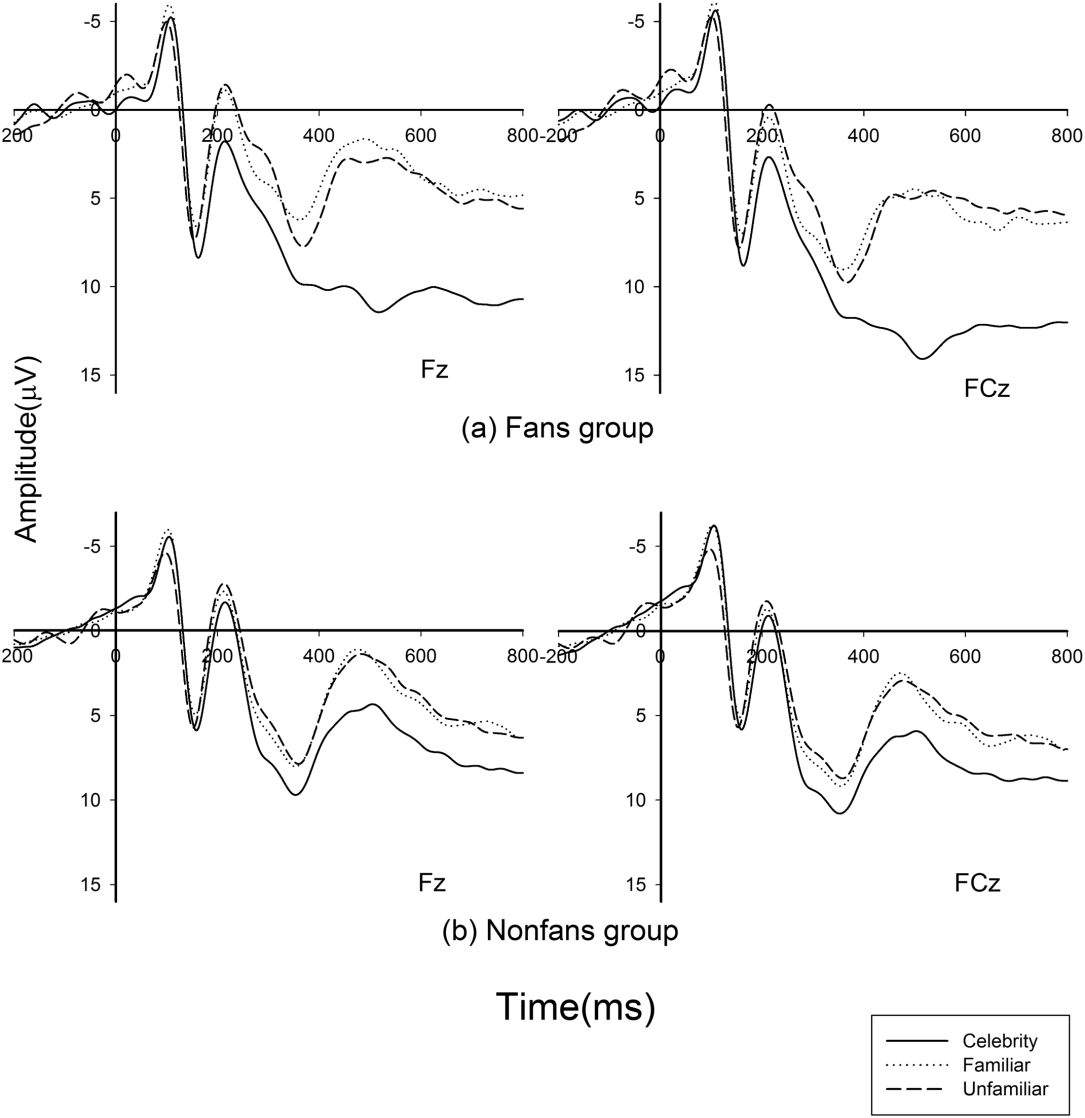
N2 results. Grand average ERP waveforms from channels Fz and FCz, which stand for the selected six electrodes as a function of the three conditions (celebrity, familiar person, and unfamiliar person) for fans (a) and non-fans (b) groups.

In the fan group, the main effect of N2 for the three conditions was significant, *F (2*, *28) = 15*.*05*, *p < 0*.*001*. The result of Bonferroni-corrected pairwise comparisons showed a significant difference between celebrity and familiar person, *p< 0*.*05*, celebrity and unfamiliar person, *p < 0*.*001*, but not between familiar and unfamiliar people, *p > 0*.*5*. The celebrity’s face (3.50 6μV) elicited smaller N2 (negative polarity, smaller voltage means larger amplitude) mean amplitude than familiar (0.922 μV) and unfamiliar (0.422 μV) faces in the fan group, as shown in Fig 1(a). Meanwhile, in the non-fan group, the main effect of the conditions was not significant, *F (2*, *28) = 2*.*495*, *p > 0*.*05*, as shown in Fig 1(b).

#### P300 results

In P300 analysis, the main effect of face was significant, *F (2*, *56) = 35*.*70*, *p < 0*.*001*. The main effect of subject group was also significant, *F (1*, *28) = 10*.*11*, *p < 0*.*04*. The fan group (9.76 μV) had larger P300 (positive polarity, larger voltage means larger amplitude) amplitude than the non-fan group (5.80 μV). The interaction effect of conditions and subject group was also significant, *F (2*, *56) = 13*.*72*, *p < 0*.*001*. Therefore, further simple effect analysis was conducted in each subject group.

**Fig 2:**
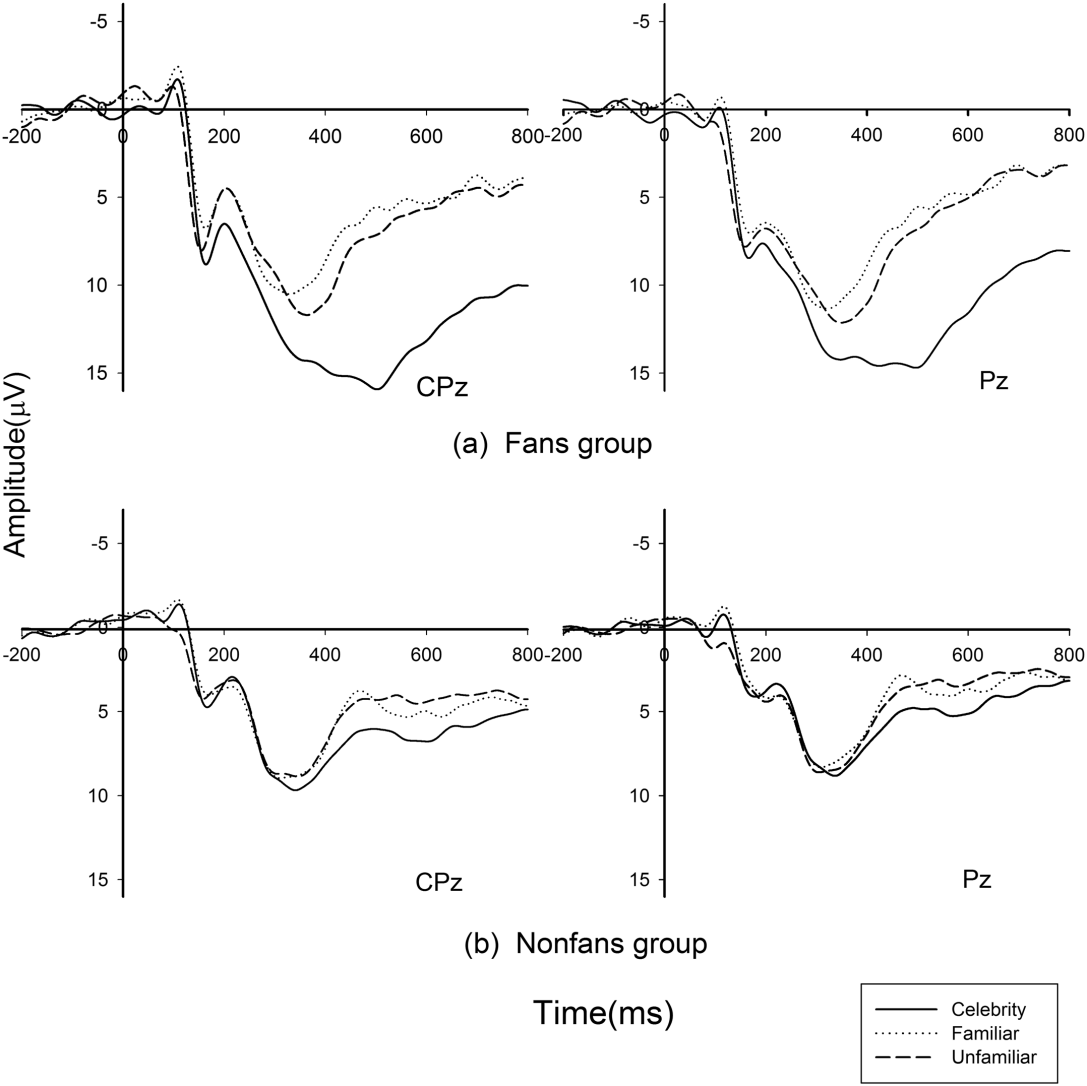
P300 results. Grand average ERP waveforms from channels CPz and Pz, which stand for the selected six electrodes as a function of the three conditions (celebrity, familiar person, and unfamiliar person) for fans (a) and non-fans (b) groups.

In the fan group, the main effect of different faces was observed, *F (2*, *28) = 33*.*53*, *p < 0*.*001*. The Bonferroni-corrected pairwise results showed that the amplitude of celebrity was significantly different from familiar, *p < 0*.*001*, and unfamiliar person, *p < 0*.*001*, whereas no difference between familiar and unfamiliar faces was observed, *p > 0*.*05*. The celebrity (13.42 μV) elicited larger P300 amplitude than the familiar (7.15 μV) and unfamiliar (8.73 μV). In the non-fan group, the main effect of the three conditions was significant, *F (2*, *28) = 4*.*43*, *p < 0*.*05*, *ε = 0*.*704*. However, the pairwise comparisons did not indicate a difference between celebrity and unfamiliar face, *p > 0*.*05*; between celebrity and familiar person, *p > 0*.*05*; and between familiar and unfamiliar faces, *p > 0*.*5*. The celebrity (6.70 μV) elicited larger P300 amplitude than the unfamiliar (5.38 μV) and familiar (5.32 μV) for the non-fan group. Fig 2 shows the two groups’ P300 waveform.

#### Celebrity Appeal Questionnaire results

The CAS scores were largely significantly different between groups in all the three dimensions. In the intense-personal dimension, the fans (50.07 ± 9.98; 37.80 ± 4.678; 17.53 ± 5.027) attained a higher score than that of the non-fans (19.80 ± 5.61; 19.00 ± 7.76; 10.60 ± 3.66), *t (28) = 10*.*24*, *p < 0*.*001*, as well as in the entertainment-social, *t (28) = 4*.*32*, *p< 0*.*001*, and borderline-pathological dimension, *t (28) = 8*.*03*, *p< 0*.*001*.

**Fig 3:**
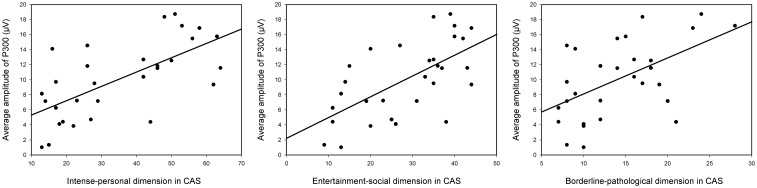
Corrections between the three dimensions in the CAS scale and average amplitude of P300.

Analyses to examine the relationship between ERP components and CAS scores in the three dimensions were conducted for P300. Pearson correlation was conducted between each of the three dimensions and average amplitude of P300 induced by celebrity’s face in all the subjects. The results showed that all three dimensions were positively correlated with the mean amplitudes of P300, as shown in Table 1 and Fig 3. The results indicate that the more the subjects like the celebrity, the larger P300 amplitude elicited by the celebrity’s photos.

**Table 1:** Correlation indexes and *p* values between the three CAS dimensions (intense-personal, entertainment-social and borderline-pathological) and average voltages of P300.

		intense-personal	entertainment-social	borderline-pathological
P300	r	.658	.630	.532
	*p*	< .001	< .001	.002

## Discussion

The purpose of the current study was to explore the neural foundation of fandom and try to measure individual involvement to a specific celebrity on the neurological level by comparing subjects’ neural responses to photos of a celebrity, a familiar person, and an unfamiliar person between groups. We also investigated the correlation between brain activity and score of the classical CAS to further confirm the validity of neural index. The statistic results of N2 component showed the main effect of different faces and interaction effect between subject group and face. Further analysis showed that, in the fan group, the celebrity face elicited smaller N2 amplitude than the faces of the familiar person and stranger, whereas in the non-fan group, the main effect of N2 was not observed in the three conditions.

As previously mentioned, the N2 component might reflect aspects of cognitive control, mismatch, or facilitate one’s actions [10]. The N2’s association with cognitive control and mismatch processes manifested as enhanced negativity to non-target stimuli, indexing the conflict between the coming stimulus and the excepted one. Thus, compared with the celebrity’s photos, the larger N2 observed in response to familiar and unfamiliar people’s photos in the fan group might reflect the inhibition of action due to fans’ anticipation of their favorite celebrity’s photo. We suspected that for their specific emotion to the celebrity, fans might also designate the celebrity as another target stimulus even though a target was present and no overt action was expected. These results were similar to Grasso’s (2009) work, in which a mother subconsciously regarded her own child’s face as a target compared with other children and adults. Such a reaction elicited smaller N2 component when processing the face of her own child even if no action was expected [8]. However, for non-fans, their emotion to celebrity was not so different from that to the familiar person and stranger.

The analysis of the later positive component showed that compared with non-fans, fans elicited larger P300 amplitudes when viewing the photo of the celebrity. Furthermore, the celebrity’s photos elicited robust P300 amplitudes compared with the other two kinds of photos in the fan group. However, in the non-fan group, the celebrity’s faces did not elicit larger P300 amplitude than other faces. Previous studies have shown that temporal–parietal P300 components could reflect attentional allocation, which can be modulated by affective processing to stimulus [16, 17, 23]. Therefore, we suspected that fans had strong affection for their favorite celebrity and paid considerable attention to the photographs of the celebrity despite their not having met the celebrity, as reflected in P300 amplitude. This result was also supported by the substantial positive correlation between P300 response to the celebrity and CAS score, suggesting the photograph of the celebrity that elicited a more positive attitude toward them will result in larger P300 amplitude. However, in the current result, we did not found any significant difference of early components between groups. Therefore, we suspected that the cognitive process of people’s attitude toward a specific celebrity was a process of emotion regulation rather than emotion arousal.

## Conclusions

In summary, by employing electrophysiological tools, the present study revealed several pieces of electrophysiological evidence for people’s involvement with a celebrity. As shown by the amplitude deflection of N2 and P300 discrepancy between groups, the celebrity gained more attention from fans than from non-fans because of the latter’s emotional involvement toward their favorite celebrity. The current study also showed the neuroscience evidence that the attitude of fans toward their favorite celebrity was similar to that for their loved child, romantic partner, or other familiar loved ones, rather than the learned familiar person. However, the celebrity was actually a learned familiar person because the fans and the celebrity have, for the most part, not met each other. Finally, brain activity could be an index to reflect whether a person is a fan of a specific celebrity.

## Supporting Information

S1 Dataset Data of N2, P300 amplitude and CAQ questionnaire. (ZIP)
